# Supplementary material for: Pirin does not bind to p65 or regulate NFκB-dependent gene expression but does modulate cellular quercetin levels
Source: bioRxiv. 2024 Dec 4:2024.12.03.626411. Preprint. [Version 1] doi: 10.1101/2024.12.03.626411 (PMC11642861; doi:10.1101/2024.12.03.626411)
Supplement: Supplement 1 [file media-1.pdf]

Supplemental Materials

Figure S1

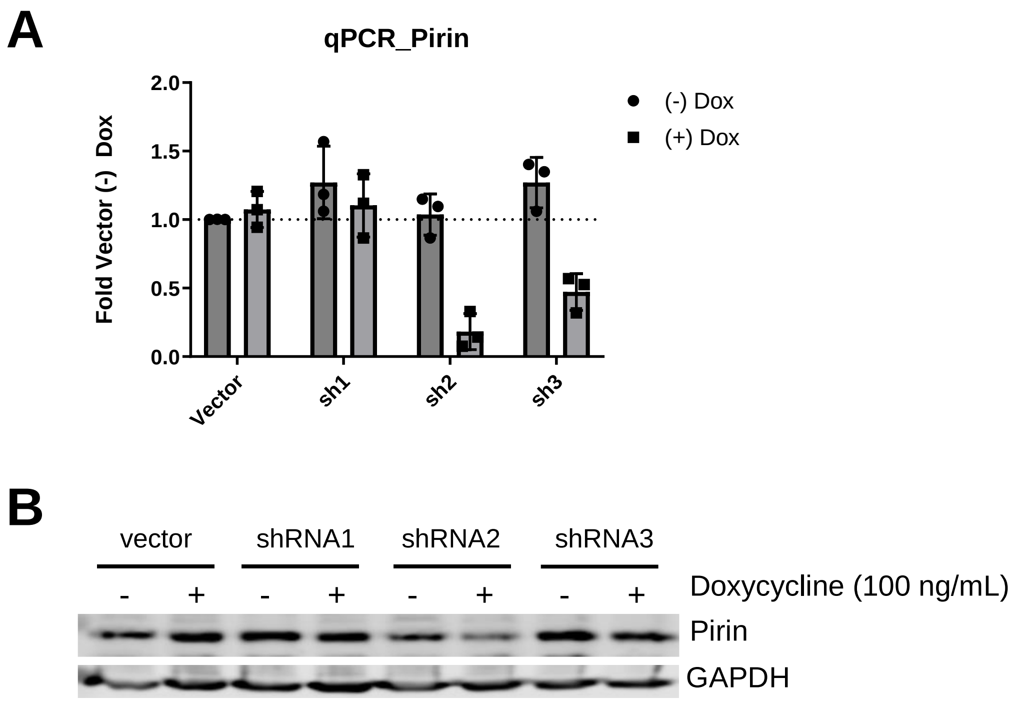

Figure S1. Generation of knockdown pirin cell lines. (A) qPCR and (B) Westernblot for pirin expression from 3T3 cells stabely transfected with different silencing RNAs for Pirin.

Figure S2

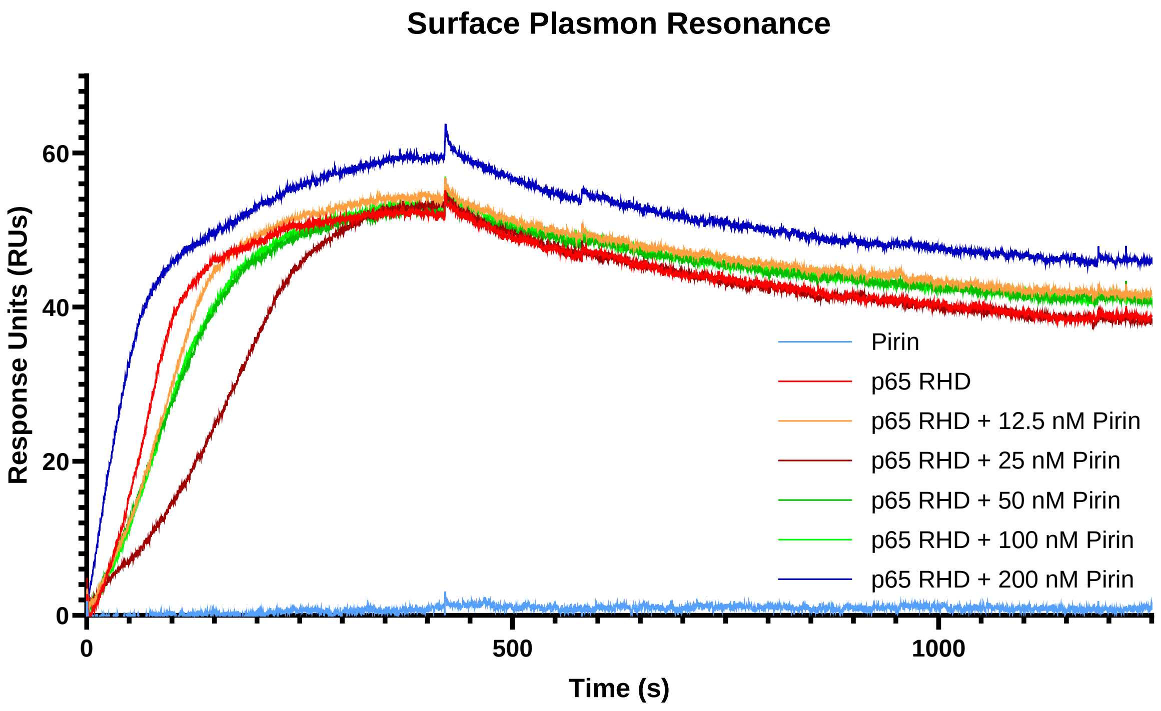

Figure S2. Surface Plasmon Resonance of p65RHR and pirin. Biotinylated IκB DNA was bound to Channel A on a Streptavidin Chip. Non-specific DNA was bound to Channel B. P65RHR alone (50 nM) or with different concentrations of Pirin (12.5 – 200 nM), or pirin alone, were run over the chip. Signal from channel B was subtracted from Channel A to correct for non-specific binding. Graph is representative of 3 different experiments.

Figure S3

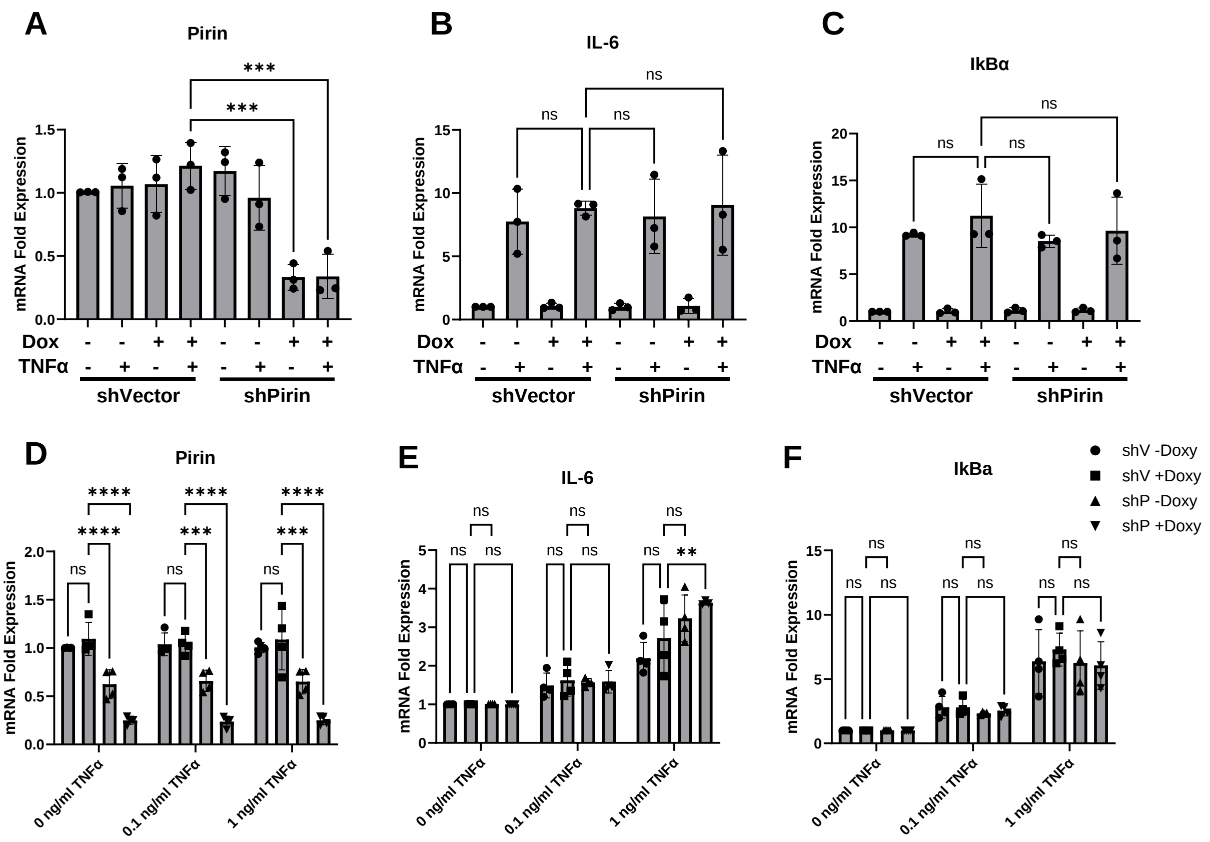

Figure S3. Expression of NF-kB response genes. (A) Full dataset shown for Figure 3. Cells were treated with 10 ng/ml TNFα. (B) shVector and shPirin cells treated with different concentrations of TNFα. Data are mean ± SD from 3 independent experiments each with 3 technical replicates. Statistical analysis was done by One-way ANOVA (A-C) or two-way ANOVA with Dunnetts (D-E) in GraphPad Prism 10 with significance values of * = p<0.05, ** = p<0.01, and *** = p<0.001.

Figure S4

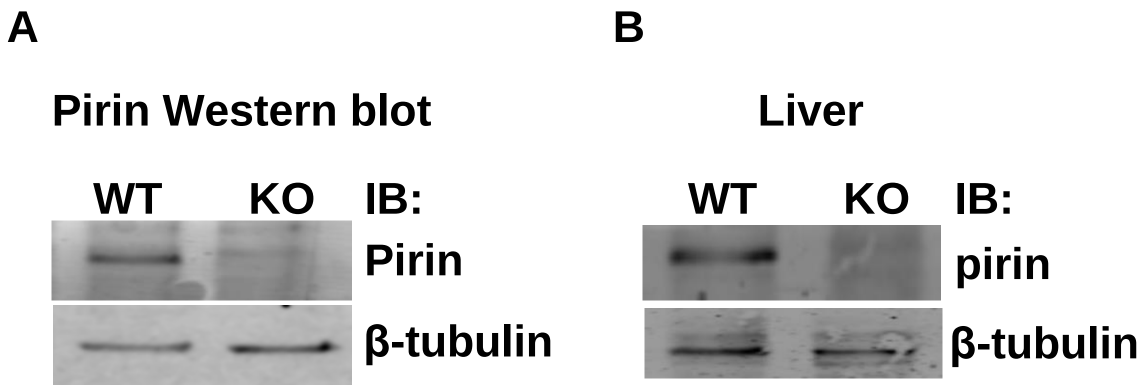

Figure S4. Pirin knockout mice show global knockout of Pirin. Westernblots show pirin protein expression in fibroblasts (A) and liver (B) of pirin knockout mice. Beta-tubulin is used as loading control.

Figure S5

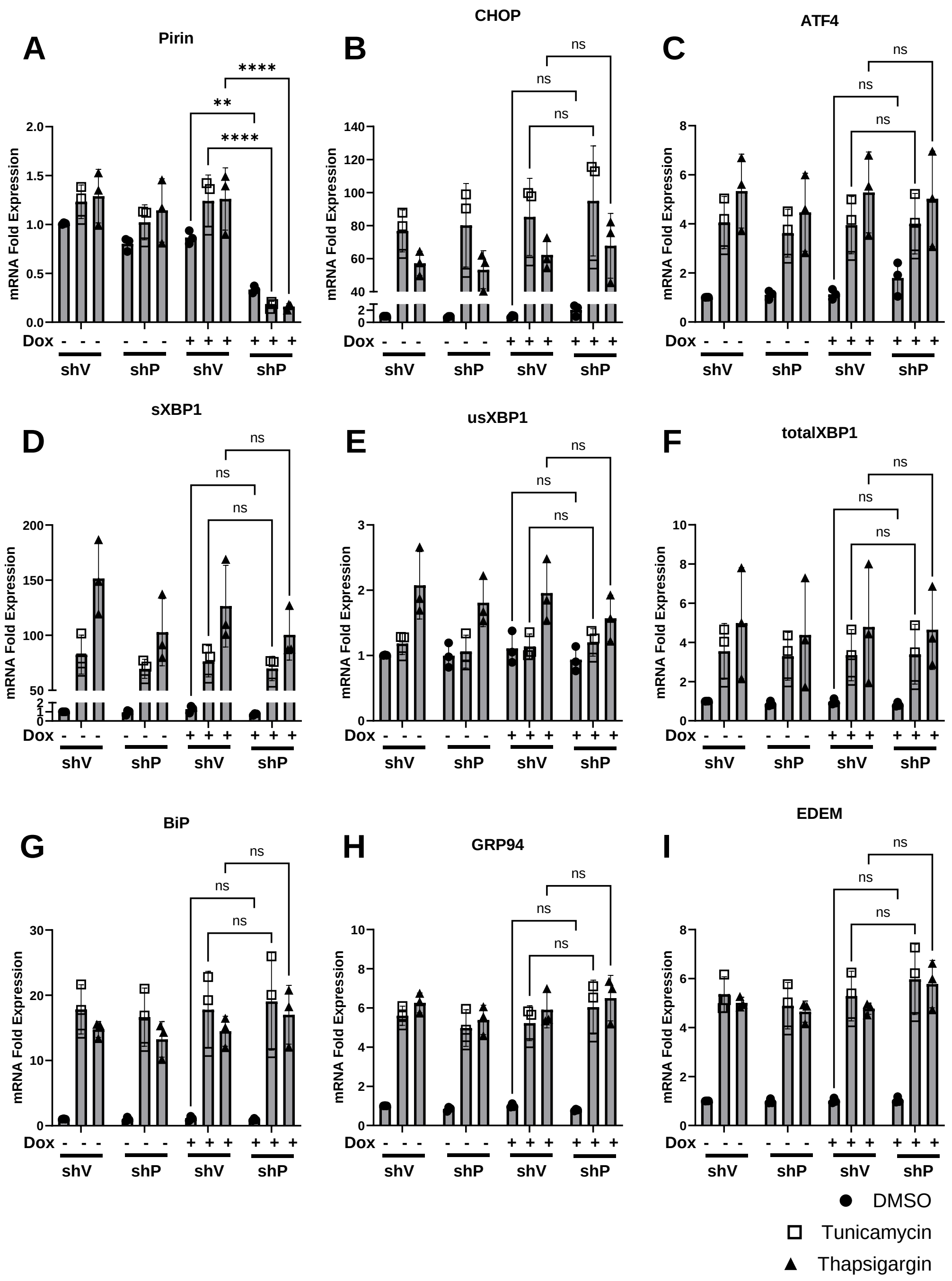

Figure S5. Expression of ER stress genes in pirin knockdown cells (shP+Dox) and control cells (shVector-Dox, shVector+Dox, shP-Dox) measured through qRT-PCR. Full

data for Figure 5. ER stress in cells was induced for 5 hours with Tunicamycin or Thapsigargin before RNA was isolated. Pirin knockdown in shP+Dox cells was confirmed through qRT-PCR (A). Statistical analysis was done by One-way ANOVA in GraphPad Prism 10 with significance values of * = $p<0.05$, ** = $p<0.01$, and *** = $p<0.001$. Error bars show standard deviation. Genes tested include CHOP (B), ATF4 (C), sXBP1 (D), usXBP1 (E), totalXBP1 (F), BiP (G), GRP94 (H), and EDEM (I).

Figure S6

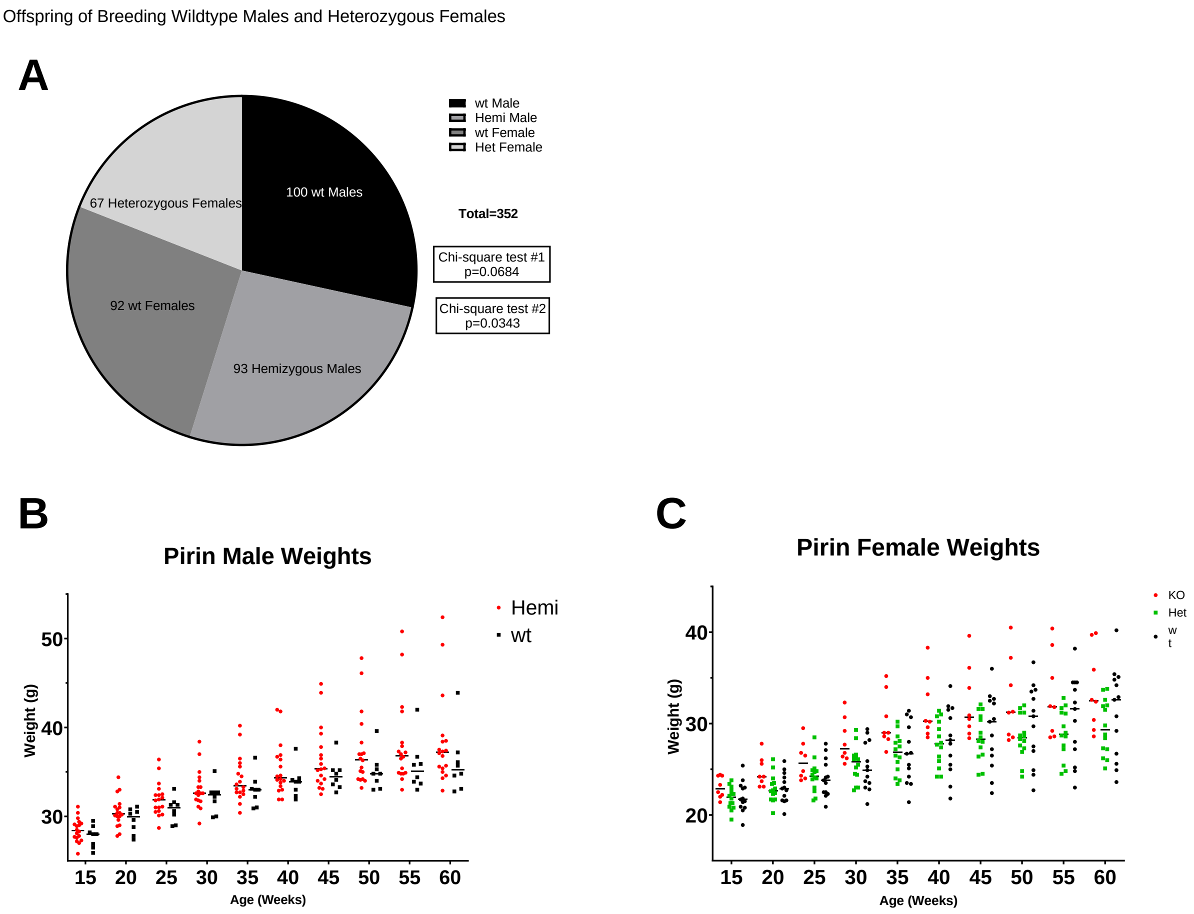

Figure S6. Breeding outcomes of Pirin knockout mice.
